# SCovid: single-cell atlases for exposing molecular characteristics of COVID-19 across 10 human tissues

**DOI:** 10.1093/nar/gkab881

**Published:** 2021-10-11

**Authors:** Changlu Qi, Chao Wang, Lingling Zhao, Zijun Zhu, Ping Wang, Sainan Zhang, Liang Cheng, Xue Zhang

**Affiliations:** College of Bioinformatics Science and Technology, Harbin Medical University, Harbin, Heilongjiang 150081, China; College of Bioinformatics Science and Technology, Harbin Medical University, Harbin, Heilongjiang 150081, China; Faculty of Computing, Harbin Institute of Technology, Harbin, Heilongjiang 150001, China; College of Bioinformatics Science and Technology, Harbin Medical University, Harbin, Heilongjiang 150081, China; College of Bioinformatics Science and Technology, Harbin Medical University, Harbin, Heilongjiang 150081, China; College of Bioinformatics Science and Technology, Harbin Medical University, Harbin, Heilongjiang 150081, China; College of Bioinformatics Science and Technology, Harbin Medical University, Harbin, Heilongjiang 150081, China; NHC and CAMS Key Laboratory of Molecular Probe and Targeted Theranostics, Harbin Medical University, Harbin, Heilongjiang 150028, China; NHC and CAMS Key Laboratory of Molecular Probe and Targeted Theranostics, Harbin Medical University, Harbin, Heilongjiang 150028, China; McKusick-Zhang Center for Genetic Medicine, Peking Union Medical College, Beijing 100005, China

## Abstract

SCovid (http://bio-annotation.cn/scovid) aims at providing a comprehensive resource of single-cell data for exposing molecular characteristics of coronavirus disease 2019 (COVID-19) across 10 human tissues. COVID-19, an epidemic caused by severe acute respiratory syndrome coronavirus 2 (SARS-CoV-2), has been found to be accompanied with multiple-organ failure since its first report in Dec 2019. To reveal tissue-specific molecular characteristics, researches regarding to COVID-19 have been carried out widely, especially at single-cell resolution. However, these researches are still relatively independent and scattered, limiting the comprehensive understanding of the impact of virus on diverse tissues. To this end, we developed a single-cell atlas of COVID-19. Firstly we collected 21 single-cell datasets of COVID-19 across 10 human tissues paired with control datasets. Then we constructed a pipeline for the analysis of these datasets to reveal molecular characteristics of COVID-19 based on manually annotated cell types. The current version of SCovid documents 1 042 227 single cells of 21 single-cell datasets across 10 human tissues, 11 713 stably expressed genes and 3778 significant differentially expressed genes (DEGs). SCovid provides a user-friendly interface for browsing, searching, visualizing and downloading all detailed information.

## INTRODUCTION

Coronavirus disease 2019 (COVID-19), caused by severe acute respiratory syndrome coronavirus 2 (SARS-CoV-2), is an ongoing global health threat since the beginning of the outbreak in late 2019 and has infected more than 190 million people worldwide as of 21 July 2021 (1). Research on isolating, sequencing and cloning the virus, development of diagnostic kits, and the testing of candidate vaccines are rapidly proceeding (2–6). However, key questions remain about the pathophysiology of COVID-19 (7).

With the in-depth case studies of COVID-19, accumulating evidence indicates that COVID-19 could not only result in acute respiratory distress syndrome but also multiorgan involvement. SARS-CoV-2 binds to angiotensin converting enzyme 2 (ACE2) receptors presented in vascular endothelial cells, lungs, heart, brain, kidneys, intestine, liver, and other tissues, which directly injures these organs (8). For example, emerging data from autopsy studies demonstrated that COVID-19 is accompanied by acute interstitial pneumonia (AIP), diffuse alveolar damage (DAD) and microvasculature involvement with pulmonary vessel hyaline thrombosis, haemorrhage, vessel wall oedema, intravascular neutrophil trapping and immune cell infiltration (9–11). In addition, gastrointestinal symptoms associated with COVID-19 vary widely but can include loss of appetite, nausea, vomiting, diarrhoea and generalized abdominal pain (12). ACE2 expression in cardiac tissue is also significantly elevated, which may potentially facilitate myocarditis caused by viral infection (13–15). To reveal tissue-specific molecular characteristics, researches regarding to COVID-19 have been carried out widely, especially at single-cell resolution. Triana et al. identified a subgroup of enterocytes as the prime target of SARS-CoV-2 and found the lack of positive correlation between infection susceptibility and ACE2 expression using single-cell RNA sequencing of SARS-CoV-2-infected colon and ileum organoids, which indicates that SARS-CoV-2 suppresses the immune response (16). Moreover, Arunachalam *et al.* revealed that various cell types exhibit unique pro- and anti-inflammatory responses by analyzing the peripheral blood mononuclear cells (PBMCs) of COVID-19 patients (17).

Since the rapid development of COVID-19 has led to the imminent researches on COVID-19, numerous COVID-19-related databases have emerged. GISAID (18), Nextstrain (19), GESS (20) and European Nucleotide Archive (21) collected SARS-CoV-2 strains of different patients all around the world and provided tools to analyse sequences. CORD-19 (22), LitCovid (23) and BioRxiv & MedRxiv summarized the literature about the latest progress in COVID-19 research. DrugBank (24), DockCoV2 (25) and COVID19 Drug Repository (26) predicted drugs with potential therapeutic effects and were well cross-linked to external databases, which provided the possibility to speed up the discovery of therapeutic drugs. Coronavirus3D (27), CoV3D (28) and RCSB PDB (29) annotated and visualized structures of coronavirus proteins and their complexes with high resolution. Besides, various types of single-cell databases such as CancerSEA (30), CellMaker (31), TISCH (32) and so on are emerging in endlessly. However, none of these databases focuses on molecular characteristics of COVID-19 patients. Therefore, we developed SCovid, a single-cell atlas for exposing molecular characteristics of COVID-19 across 10 human tissues. This database could be freely available at: http://bio-annotation.cn/scovid.

## DATA COLLECTION AND DATABASE CONTENT

We manually searched COVID-19 related single cell RNA-seq (scRNA-seq) datasets in electronic databases, including Sequence Read Archive (SRA) (33) and Gene Expression Omnibus (GEO) (34), based on the keywords: (‘COVID-19’ OR ‘SARS-CoV-2’) AND (‘single cell’ OR ‘single-cell’) AND (‘transcriptomics’ OR ‘transcriptome’ OR ‘scRNA-seq’ OR ‘scRNA seq’). Meanwhile, we also systematically searched electronic databases, including PubMed, National Library of Medicine of the National Institutes of Health, BioRxiv and MedRxiv preprint services operated by Cold Spring Harbor Laboratory, through searching for the keywords such as ‘single cell sequencing’, ‘scRNA-seq & COVID-19’ and ‘transcriptomics & COVID-19’. Literature and host data on COVID-19 were manually extracted from publications. Finally, a total of 21 COVID-19 related scRNA-seq datasets involving 10 tissue types were obtained. All datasets were collected before July 2021.

Considering the technical noise of assay, we removed low quality cells and lowly expressed genes of each COVID-19 related scRNA-seq datasets for further analysis, using the following strategy: (i) cells that had fewer than 200 genes, as well as genes expressed in fewer than three cells; (ii) liver cells that contained greater than 50% of mitochondrial genes, as well as other tissue cells that contained >20% of mitochondrial genes. For each dataset, we used the R package ‘Seurat’ (v3.2.3) (35) for data integrating, clustering, dimensionality reduction, and visualization. For these analyses, the function ‘SCTransform’ was used to integrate and scale data. Then, PCA analysis was performed using variable feature genes, and the principal components (PCs) identified by the function ‘ElbowPlot’ were used to cluster the dataset. Next, each cluster annotation was confirmed by our previous knowledge of known cell type-specific gene markers, which were obtained from DE genes of each cluster by ‘FindMarkers’ function. Subsequently, we performed UMAP to reduce the dataset into two-dimension, and finally the cells were visualized on the website. We also performed analysis of scRNA-seq expression, including DE genes and gene pathway. First, for each cell type, MAST (v1.16.0) (36) was used to calculated differentially expressed genes (DEGs) between the cells from samples with COVID-19 and the cells from controls. Then, up/down-regulated genes with top 5% |Log_2_FC| and *P* value <0.05 were regarded as significant DEGs, which were visualized by volcano plot. Next, the GO pathways of each cell type were enriched using these up/down-regulated significant DEGs by R package clusterProfiler (37).

**Figure 1.**
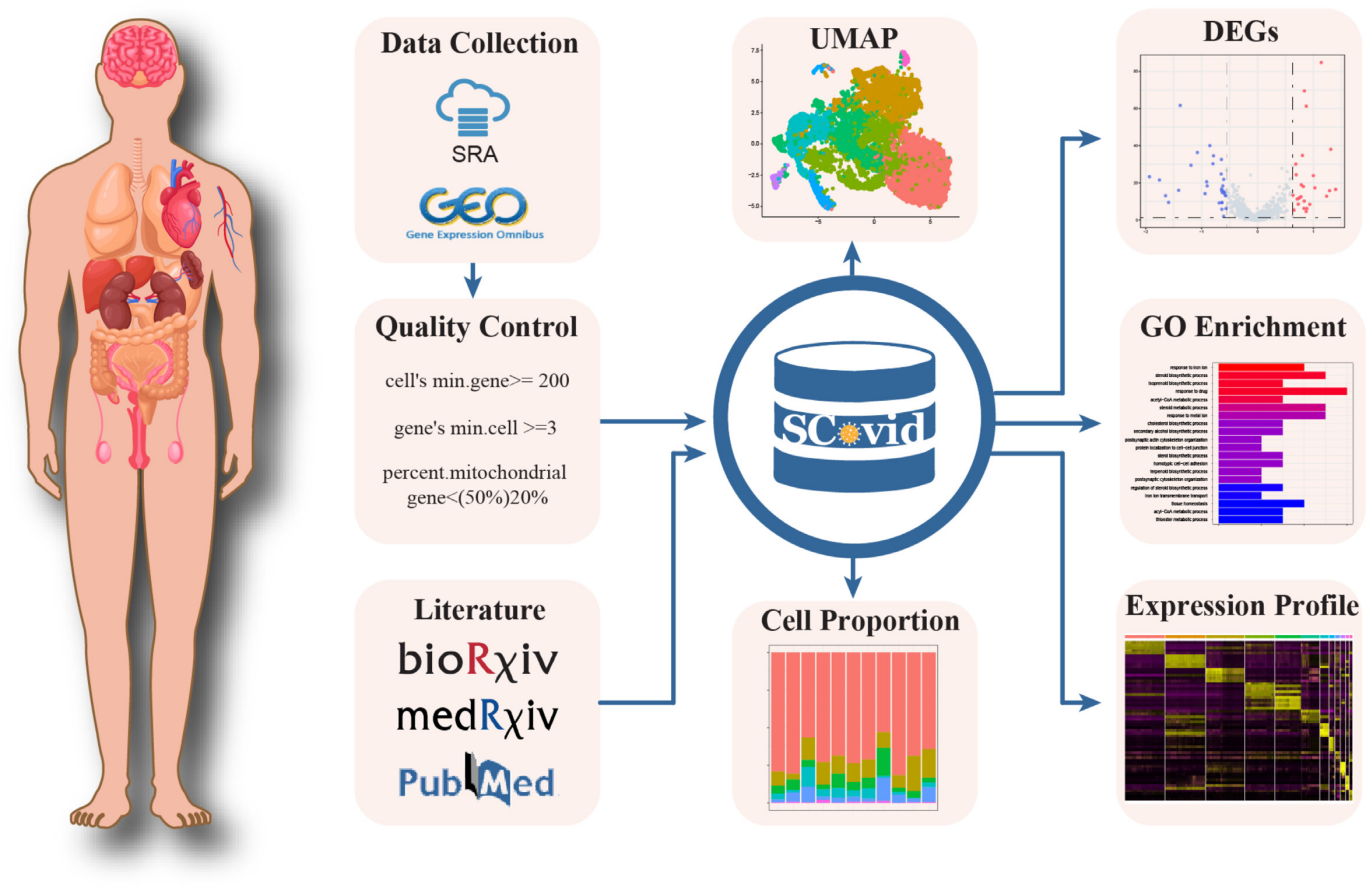
Overview of SCovid database.

**Figure 2.**
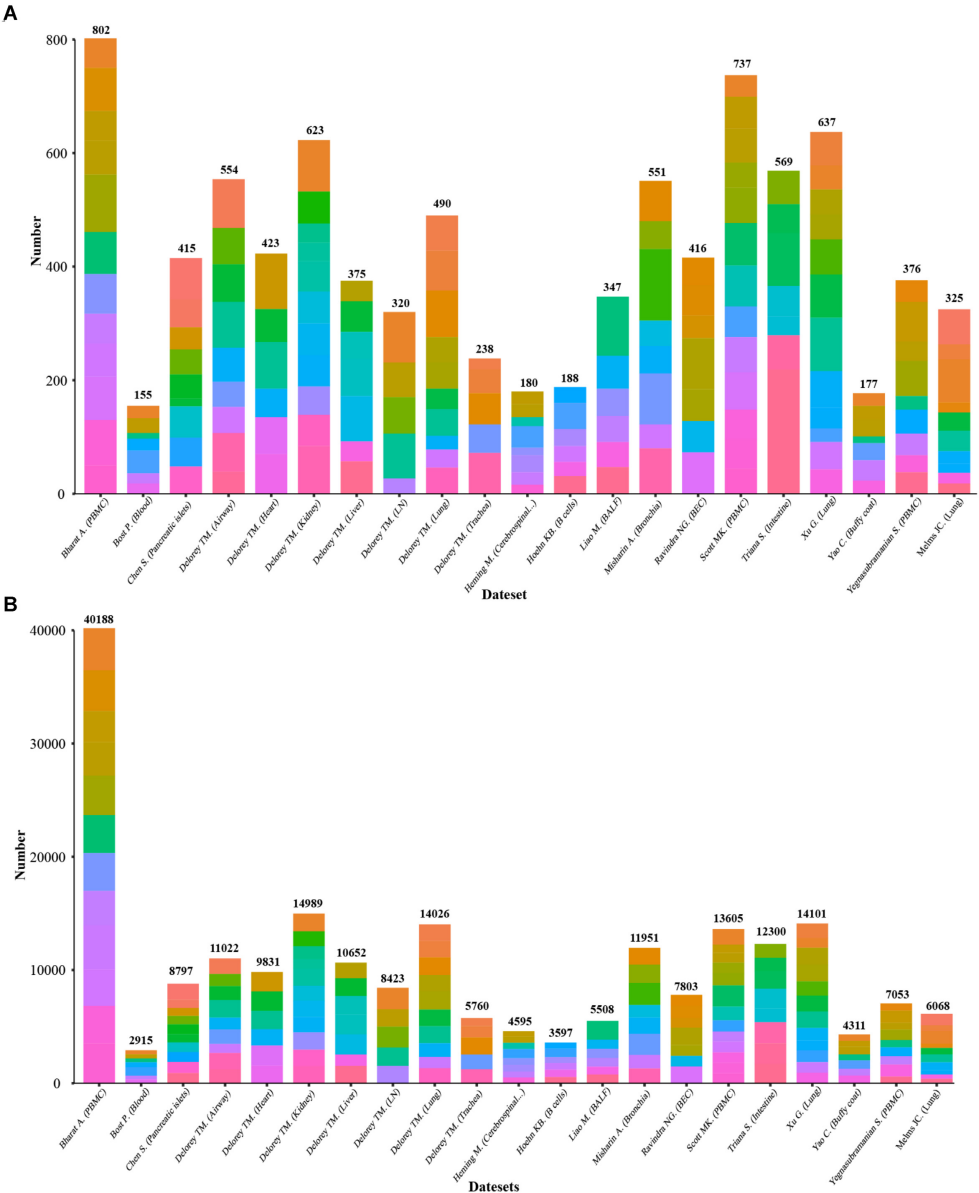
Number of genes in each dataset. Each color represents a different cell type. (**A**) Number of significant DEGs in each dataset. (**B**) Number of stably expressed genes in each dataset.

**Figure 3.**
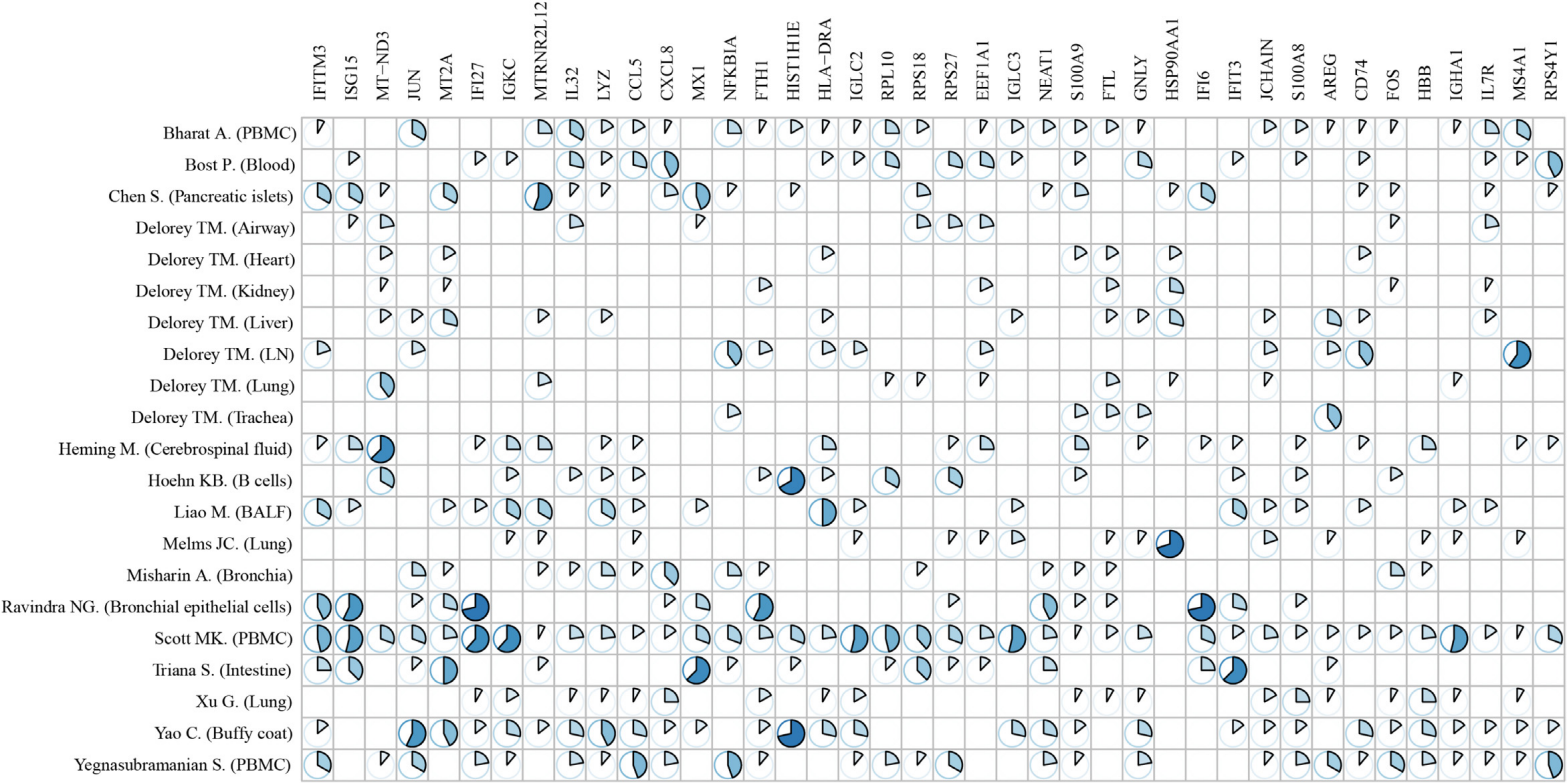
The most frequently occurring significant DEGs in cell types of these 21 datasets. The area of the sector represents the proportion of cell types where this gene is significant DEG in the dataset.

Overview of SCovid database is shown in Figure 1. The current version of SCovid documents 1 042 227 single cells of 21 single-cell datasets across 10 human tissues (including intestine, blood, pancreas, lung, brain, airway, heart, kidney, liver and lymph node), 11 713 stably expressed genes (217 495 associations) and 3778 significant DEGs (8898 associations). Each dataset in SCovid contains detailed information of data source, sample source, grouping information, single-cell number and cell types. Each entry of DEGs contains Log_2_FC, *P* value and visual information. Figure 2 shows the number of genes in each dataset. Figure 3 shows the most frequently occurred significant DEGs that might be potential cell-type specific markers in these 21 datasets.

## USER INTERFACE

**Figure 4.**
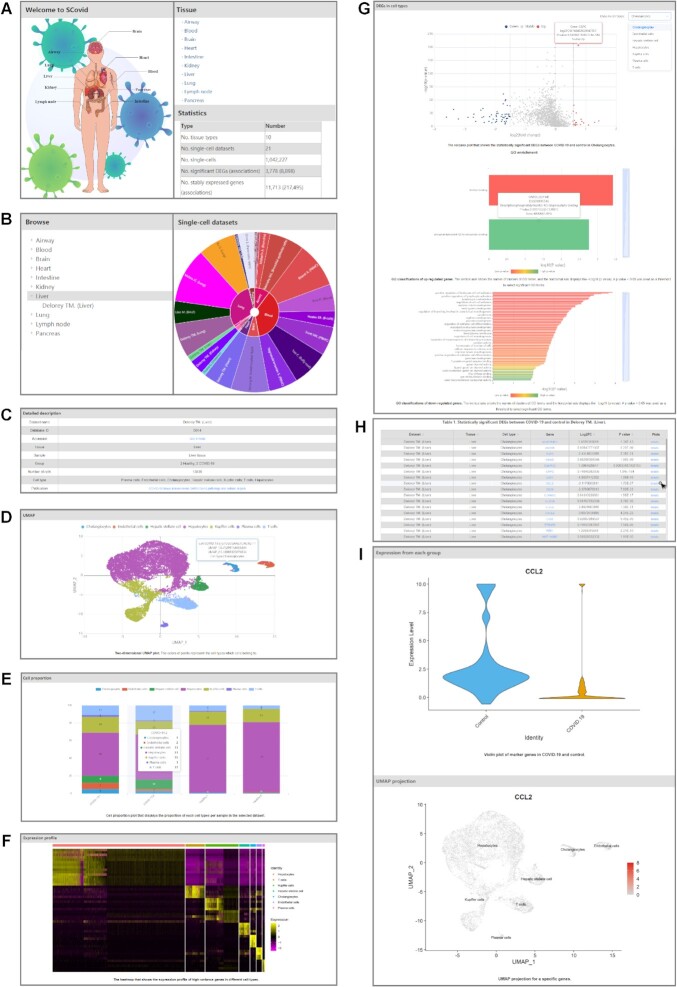
Browse page and results of SCovid. (**A**) Home page of Scovid. (**B**) The tree browser of SCovid in Browse page. (**C**) Detailed description of this dataset. (**D**) Two-dimensional UMAP plot. The colors of points represent the cell types which cells belong to. (**E**) Cell proportion plot that displays the proportion of each cell types per sample in the selected dataset. (**F**) The heatmap that shows the expression profile of high-variance genes in different cell types. (**G**) The volcano plot that shows the statistically significant DEGs between COVID-19 and control and GO enrichment bar plots of up/down-regulated. In the GO enrichment bar plots, the vertical axis shows the names of clusters of GO terms, and the horizontal axis displays the − Log_10_ (*P* value). A *P* value <0.05 was used as a threshold to select significant GO terms. (**H**) The table that shows statistically significant DEGs between COVID-19 and control. (**I**) The violin plot of a specific gene in COVID-19 and control and UMAP projection for a specific gene.

**Figure 5.**
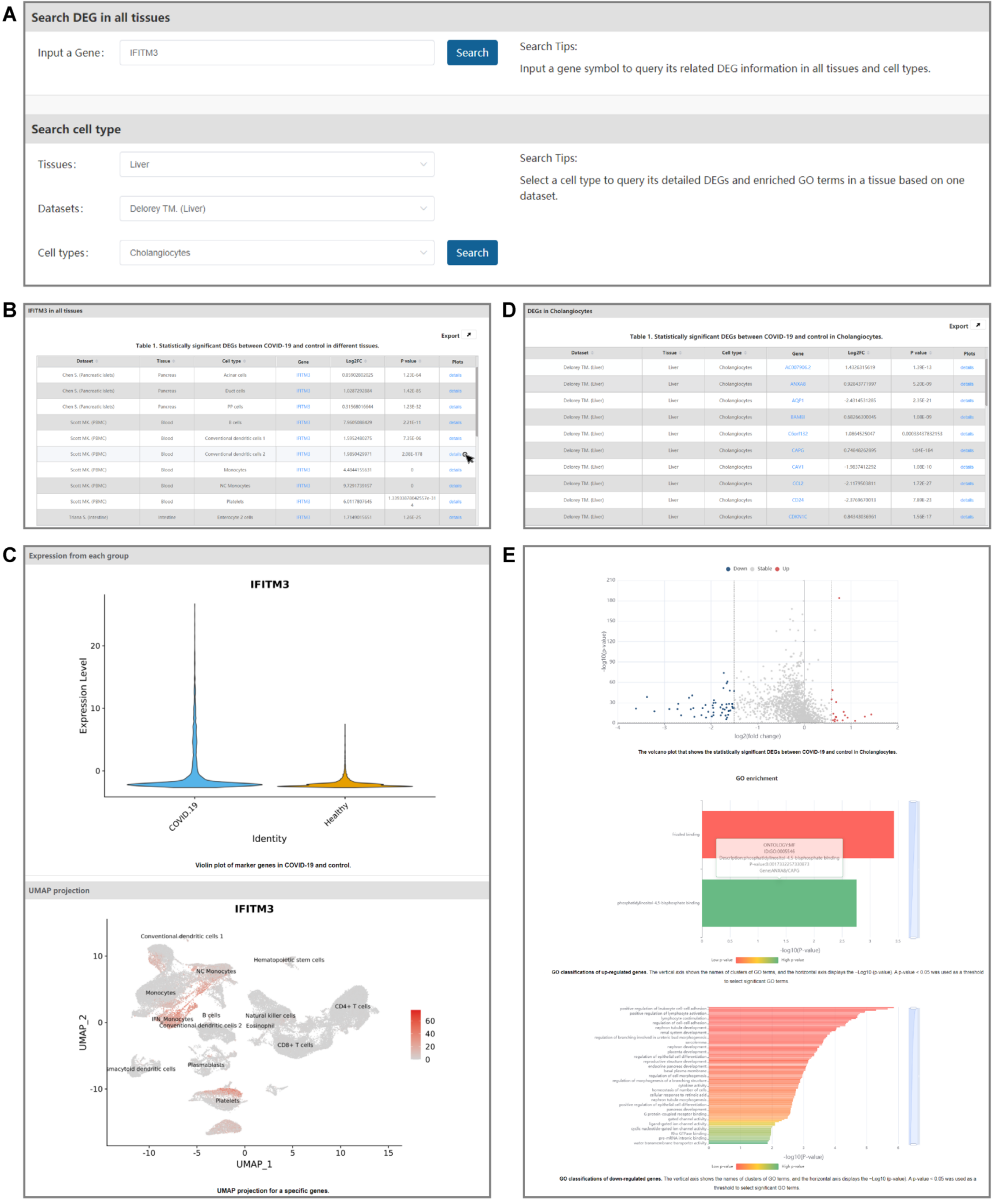
Search page and results of SCovid. (**A**) Search page of SCovid. (**B**) The table that shows statistically significant DEGs between COVID-19 and control in different tissues. (**C**) The violin plot of a specific gene in COVID-19 and control and UMAP projection for a specific gene. (**D**) The table that shows statistically significant DEGs between COVID-19 and control. (**E**) The volcano plot that shows the statistically significant DEGs between COVID-19 and control and GO enrichment bar plots of up/down-regulated. In the GO enrichment bar plots, the vertical axis shows the names of clusters of GO terms, and the horizontal axis displays the − Log_10_ (*P* value). A *P* value <0.05 was used as a threshold to select significant GO terms.

We provided a user-friendly web interface to visualize the datasets by a few flexible steps as shown in Figures 4 and 5. All datasets are organized according to tissues types. Users can browse datasets by clicking the corresponding tissue icon or ‘Tissue’ hyperlinks in the ‘Home’ page or clicking specific tissue name in the navigation menu in the ‘Browse’ page (Figure 4A and B). After selecting a dataset, for example, ‘Delorey TM. (Liver)’, all the detailed and visual information, including ‘Detailed description’, ‘UMAP’, ‘Cell proportion’, ‘DEGs in cell types’ and ‘Expression profile’, would be retrieved.

- *Detailed description*. The ‘Detailed description’ section contains dataset name, tissue type, accession number, number of cells, cell types, sample source and relevant publication information (Figure 4C). Additionally, accession number and publication title contain hyperlinks the clients can follow.
- *UMAP*. Visualization of the selected dataset using UMAP analysis is displayed in the ‘UMAP’ section with colorful points representing different cell types (Figure 4D).
- *Cell proportion*. The ‘Cell proportion’ section displays a bar plot to show the cell-type proportion across samples (Figure 4E). Each bar represents a sample and different colors represent different cell types.
- *DEGs in cell types*. In the ‘DEGs in cell types’ section, users can select the interested cell type to browse the interactive information including a volcano plot, a table and Gene ontology (GO) (38) enrichment bar plots (Figure 4G and H). When positioning the mouse on any bubbles of the volcano plot showing all stably expressed genes, the detailed information including gene symbol, Log_2_FC, *P* value and change status would be popped up. The result table is used to display the statistically significant DEGs between COVID-19 and control in the selected cell type of this dataset. In the result table, clicking the ‘detail’ link of a row would lead to the detailed plots including a violin plot and a UMAP projection plot for the specific gene (Figure 4I). The GO enrichment bar plots displaying GO classifications of up/down-regulated genes, in which hovering over any bars would pop up detailed information including ontology aspect, term ID, term description, *P* value and genes’ symbol.
- *Expression profile*. The ‘Expression profile’ section provides the heatmap that shows the expression profile of high-variance genes in different cell types (Figure 4F). The individual tiles in the heatmap are scaled with a range of colors proportionate to gene expression values. The gene sequences correspond to the rows of the matrix and the cells correspond to the columns.
- *Data search*. In the ‘Search’ page, SCovid offers two sections involving ‘Search DEG in all tissues’ and ‘Search cell type’ (Figure 5A). For a gene, SCovid allows users to input its symbol to query its related DEG information in all tissues and cell types and a table will be returned as described above on the Browse page (Figure 5B and C). By selecting cell type, users will query its detailed DEGs and enriched GO terms in a tissue based on one dataset (Figure 5D and E).
- *Data download*. In addition, all data in SCovid can be downloaded in the ‘Download’ page, containing the DEGs' expression profile, variation information of all stably expressed genes and DEGs.

## SUMMARY AND FUTURE PERSPECTIVES

Since the outbreak of COVID-19 in Dec. 2019, databases about the literature collection, SARS-CoV-2 genome sequencing or proteins’ structures, and drug prediction appeared subsequently, while none of them focuses on molecular characteristics of COVID-19 patients. Given the high accuracy and cellular specificity of single-cell sequencing, we collected 21 single-cell datasets of COVID-19 across 10 human tissues paired with control datasets to reveal molecular characteristics of COVID-19 based on manually annotated cell types. We further developed a database system SCovid to provide a user-friendly interface for browsing, searching, visualizing and downloading stably expressed genes, significant DEGs and functional analysis of these significant DEGs based on cell types across tissues. The current version of SCovid documents 1 042 227 single cells of 21 single-cell datasets across 10 human tissues, 11 713 stably expressed genes and 3778 significant DEGs. Each dataset in the SCovid contains detailed information of data source, sample source, grouping information, single-cell number and cell types. Each entry of DEGs contains Log_2_FC, *P* value and visual information.

SCovid is a powerful and high-quality database for molecular characteristics of COVID-19. Biologist can access the variation information of genes of interest on specific cell types of different tissues, and the enrichment pathways of differential genes on specific cell types of different tissues. Bioinformatician can use machine learning methods to predict tissue-specific driver genes and therapeutic drugs of COVID-19. Although there is limited single-cell data of COVID-19 currently, research on COVID-19 will increase largely, since there is no effective way to completely inhibit the spread of the virus now. Meanwhile, the research focus has gradually shifted from virus strains to molecular characteristics of COVID-19 patients, which means genomics, epigenomics and proteinomics data of COVID-19 will continue to emerge. Therefore, we will focus continuously on the latest data and construct unified analysis pipelines, so as to continuously update our database.

## DATA AVAILABILITY

This database could be freely available at: http://bio-annotation.cn/scovid. The code is available at https://github.com/ChangluQi/scovid.

## References

[1] Jiang F. , Deng L., Zhang L., Cai Y., Cheung C.W., Xia Z. Review of the clinical characteristics of coronavirus disease 2019 (COVID-19). J. Gen. Intern. Med. 2020; 35:1545–1549. 32133578 10.1007/s11606-020-05762-w PMC7088708

[2] Kim D. , Lee J.Y., Yang J.S., Kim J.W., Kim V.N., Chang H. The architecture of SARS-CoV-2 transcriptome. Cell. 2020; 181:914–921. 32330414 10.1016/j.cell.2020.04.011 PMC7179501

[3] Wang C. , Liu Z., Chen Z., Huang X., Xu M., He T., Zhang Z. The establishment of reference sequence for SARS-CoV-2 and variation analysis. J. Med. Virol. 2020; 92:667–674. 32167180 10.1002/jmv.25762 PMC7228400

[4] van Kasteren P.B. , van der Veer B., van den Brink S., Wijsman L., de Jonge J., van den Brandt A., Molenkamp R., Reusken C., Meijer A. Comparison of seven commercial RT-PCR diagnostic kits for COVID-19. J. Clin. Virol. 2020; 128:104412. 32416600 10.1016/j.jcv.2020.104412 PMC7206434

[5] Dai L. , Gao G.F. Viral targets for vaccines against COVID-19. Nat. Rev. Immunol. 2021; 21:73–82. 33340022 10.1038/s41577-020-00480-0 PMC7747004

[6] Cheng L. , Han X., Zhu Z., Qi C., Wang P., Zhang X. Functional alterations caused by mutations reflect evolutionary trends of SARS-CoV-2. Brief. Bioinform. 2021; 22:1442–1450. 33580783 10.1093/bib/bbab042 PMC7953981

[7] Bohn M.K. , Hall A., Sepiashvili L., Jung B., Steele S., Adeli K. Pathophysiology of COVID-19: mechanisms underlying disease severity and progression. Physiology (Bethesda). 2020; 35:288–301. 32783610 10.1152/physiol.00019.2020 PMC7426542

[8] Shang J. , Ye G., Shi K., Wan Y., Luo C., Aihara H., Geng Q., Auerbach A., Li F. Structural basis of receptor recognition by SARS-CoV-2. Nature. 2020; 581:221–224. 32225175 10.1038/s41586-020-2179-y PMC7328981

[9] Xu Z. , Shi L., Wang Y., Zhang J., Huang L., Zhang C., Liu S., Zhao P., Liu H., Zhu L. et al. Pathological findings of COVID-19 associated with acute respiratory distress syndrome. Lancet Respir. Med. 2020; 8:420–422. 32085846 10.1016/S2213-2600(20)30076-X PMC7164771

[10] Buja L.M. , Wolf D.A., Zhao B., Akkanti B., McDonald M., Lelenwa L., Reilly N., Ottaviani G., Elghetany M.T., Trujillo D.O. et al. The emerging spectrum of cardiopulmonary pathology of the coronavirus disease 2019 (COVID-19): Report of 3 autopsies from Houston, Texas, and review of autopsy findings from other United States cities. Cardiovasc. Pathol. 2020; 48:107233. 32434133 10.1016/j.carpath.2020.107233 PMC7204762

[11] Barton L.M. , Duval E.J., Stroberg E., Ghosh S., Mukhopadhyay S. COVID-19 autopsies, oklahoma, USA. Am. J. Clin. Pathol. 2020; 153:725–733. 32275742 10.1093/ajcp/aqaa062 PMC7184436

[12] Luo S. , Zhang X., Xu H. Don’t overlook digestive symptoms in patients with 2019 novel coronavirus disease (COVID-19). Clin. Gastroenterol. Hepatol. 2020; 18:1636–1637. 32205220 10.1016/j.cgh.2020.03.043 PMC7154217

[13] Gembardt F. , Sterner-Kock A., Imboden H., Spalteholz M., Reibitz F., Schultheiss H.P., Siems W.E., Walther T. Organ-specific distribution of ACE2 mRNA and correlating peptidase activity in rodents. Peptides. 2005; 26:1270–1277. 15949646 10.1016/j.peptides.2005.01.009 PMC7115528

[14] Corrigendum to: Coronavirus fulminant myocarditis saved with glucocorticoid and human immunoglobulin. Eur. Heart J. 2021; 42:191. 32236443 10.1093/eurheartj/ehaa248 PMC7813621

[15] Inciardi R.M. , Lupi L., Zaccone G., Italia L., Raffo M., Tomasoni D., Cani D.S., Cerini M., Farina D., Gavazzi E. et al. Cardiac involvement in a patient with coronavirus disease 2019 (COVID-19). JAMA Cardiol. 2020; 5:819–824. 32219357 10.1001/jamacardio.2020.1096 PMC7364333

[16] Triana S. , Metz-Zumaran C., Ramirez C., Kee C., Doldan P., Shahraz M., Schraivogel D., Gschwind A.R., Sharma A.K., Steinmetz L.M. et al. Single-cell analyses reveal SARS-CoV-2 interference with intrinsic immune response in the human gut. Mol. Syst. Biol. 2021; 17:e10232. 33904651 10.15252/msb.202110232 PMC8077299

[17] Arunachalam P.S. , Wimmers F., Mok C.K.P., Perera R., Scott M., Hagan T., Sigal N., Feng Y., Bristow L., Tak-Yin Tsang O. et al. Systems biological assessment of immunity to mild versus severe COVID-19 infection in humans. Science. 2020; 369:1210–1220. 32788292 10.1126/science.abc6261 PMC7665312

[18] Shu Y. , McCauley J. GISAID: Global initiative on sharing all influenza data - from vision to reality. Euro Surveill. 2017; 22:30494. 28382917 10.2807/1560-7917.ES.2017.22.13.30494 PMC5388101

[19] Hadfield J. , Megill C., Bell S.M., Huddleston J., Potter B., Callender C., Sagulenko P., Bedford T., Neher R.A. Nextstrain: real-time tracking of pathogen evolution. Bioinformatics. 2018; 34:4121–4123. 29790939 10.1093/bioinformatics/bty407 PMC6247931

[20] Fang S. , Li K., Shen J., Liu S., Liu J., Yang L., Hu C.D., Wan J. GESS: a database of global evaluation of SARS-CoV-2/hCoV-19 sequences. Nucleic Acids Res. 2021; 49:D706–D714. 33045727 10.1093/nar/gkaa808 PMC7778918

[21] Amid C. , Alako B.T.F., Balavenkataraman Kadhirvelu V., Burdett T., Burgin J., Fan J., Harrison P.W., Holt S., Hussein A., Ivanov E. et al. The european nucleotide archive in 2019. Nucleic Acids Res. 2020; 48:D70–D76. 31722421 10.1093/nar/gkz1063 PMC7145635

[22] Lu Wang L. , Lo K., Chandrasekhar Y., Reas R., Yang J., Eide D., Funk K., Kinney R., Liu Z., Merrill W. et al. CORD-19: the Covid-19 open research dataset. 2020; arXiv doi:10 July 2020, preprint: not peer reviewed https://arxiv.org/abs/2004.10706v2.

[23] Chen Q. , Allot A., Lu Z. LitCovid: an open database of COVID-19 literature. Nucleic Acids Res. 2021; 49:D1534–D1540. 33166392 10.1093/nar/gkaa952 PMC7778958

[24] Wishart D.S. , Feunang Y.D., Guo A.C., Lo E.J., Marcu A., Grant J.R., Sajed T., Johnson D., Li C., Sayeeda Z. et al. DrugBank 5.0: a major update to the DrugBank database for 2018. Nucleic Acids Res. 2018; 46:D1074–D1082. 29126136 10.1093/nar/gkx1037 PMC5753335

[25] Chen T.F. , Chang Y.C., Hsiao Y., Lee K.H., Hsiao Y.C., Lin Y.H., Tu Y.E., Huang H.C., Chen C.Y., Juan H.F. DockCoV2: a drug database against SARS-CoV-2. Nucleic Acids Res. 2021; 49:D1152–D1159. 33035337 10.1093/nar/gkaa861 PMC7778986

[26] Tworowski D. , Gorohovski A., Mukherjee S., Carmi G., Levy E., Detroja R., Mukherjee S.B., Frenkel-Morgenstern M. COVID19 Drug Repository: text-mining the literature in search of putative COVID19 therapeutics. Nucleic Acids Res. 2021; 49:D1113–D1121. 33166390 10.1093/nar/gkaa969 PMC7778969

[27] Sedova M. , Jaroszewski L., Alisoltani A., Godzik A. Coronavirus3D: 3D structural visualization of COVID-19 genomic divergence. Bioinformatics. 2020; 36:4360–4362. 32470119 10.1093/bioinformatics/btaa550 PMC7314196

[28] Gowthaman R. , Guest J.D., Yin R., Adolf-Bryfogle J., Schief W.R., Pierce B.G. CoV3D: a database and resource for high resolution coronavirus protein structures. Nucleic Acids Res. 2021; 49:D282–D287. 32890396 10.1093/nar/gkaa731 PMC7778948

[29] Rose Y. , Duarte J.M., Lowe R., Segura J., Bi C., Bhikadiya C., Chen L., Rose A.S., Bittrich S., Burley S.K. et al. RCSB Protein Data Bank: Architectural advances towards integrated searching and efficient access to macromolecular structure data from the PDB Archive. J. Mol. Biol. 2021; 433:166704. 33186584 10.1016/j.jmb.2020.11.003 PMC9093041

[30] Yuan H. , Yan M., Zhang G., Liu W., Deng C., Liao G., Xu L., Luo T., Yan H., Long Z. et al. CancerSEA: a cancer single-cell state atlas. Nucleic Acids Res. 2019; 47:D900–D908. 30329142 10.1093/nar/gky939 PMC6324047

[31] Zhang X. , Lan Y., Xu J., Quan F., Zhao E., Deng C., Luo T., Xu L., Liao G., Yan M. et al. CellMarker: a manually curated resource of cell markers in human and mouse. Nucleic Acids Res. 2019; 47:D721–D728. 30289549 10.1093/nar/gky900 PMC6323899

[32] Sun D. , Wang J., Han Y., Dong X., Ge J., Zheng R., Shi X., Wang B., Li Z., Ren P. et al. TISCH: a comprehensive web resource enabling interactive single-cell transcriptome visualization of tumor microenvironment. Nucleic Acids Res. 2021; 49:D1420–D1430. 33179754 10.1093/nar/gkaa1020 PMC7778907

[33] Leinonen R. , Sugawara H., Shumway M. International Nucleotide Sequence Database, C. The sequence read archive. Nucleic Acids Res. 2011; 39:D19–D21. 21062823 10.1093/nar/gkq1019 PMC3013647

[34] Clough E. , Barrett T. The gene expression omnibus database. Methods Mol. Biol. 2016; 1418:93–110. 27008011 10.1007/978-1-4939-3578-9_5 PMC4944384

[35] Butler A. , Hoffman P., Smibert P., Papalexi E., Satija R. Integrating single-cell transcriptomic data across different conditions, technologies, and species. Nat. Biotechnol. 2018; 36:411–420. 29608179 10.1038/nbt.4096 PMC6700744

[36] Finak G. , McDavid A., Yajima M., Deng J., Gersuk V., Shalek A.K., Slichter C.K., Miller H.W., McElrath M.J., Prlic M. et al. MAST: a flexible statistical framework for assessing transcriptional changes and characterizing heterogeneity in single-cell RNA sequencing data. Genome Biol. 2015; 16:278. 26653891 10.1186/s13059-015-0844-5 PMC4676162

[37] Yu G. , Wang L.G., Han Y., He Q.Y. clusterProfiler: an R package for comparing biological themes among gene clusters. OMICS. 2012; 16:284–287. 22455463 10.1089/omi.2011.0118 PMC3339379

[38] Ashburner M. , Ball C.A., Blake J.A., Botstein D., Butler H., Cherry J.M., Davis A.P., Dolinski K., Dwight S.S., Eppig J.T. et al. Gene ontology: tool for the unification of biology. The gene ontology consortium. Nat. Genet. 2000; 25:25–29. 10802651 10.1038/75556 PMC3037419

